# Complete Genome Sequence of a Novel Reassortant Avian Influenza H1N2 Virus Isolated from a Domestic Sparrow in 2012

**DOI:** 10.1128/genomeA.00431-13

**Published:** 2013-07-18

**Authors:** Zhixun Xie, Jie Guo, Liji Xie, Jiabo Liu, Yaoshan Pang, Xianwen Deng, Zhiqin Xie, Qing Fan, Sisi Luo

**Affiliations:** Guangxi Key Laboratory of Animal Vaccines and Diagnostics, Guangxi Veterinary Research Institute, Nanning, Guangxi Province, China

## Abstract

We report here the complete genome sequence of a novel H1N2 avian influenza virus strain, A/Sparrow /Guangxi/GXs-1/2012 (H1N2), isolated from a sparrow in the Guangxi Province of southern China in 2012. All of the 8 gene segments (hemagglutinin [HA], nucleoprotein [NP], matrix [M], polymerase basic 2 [PB2], neuraminidase [NA], polymerase acidic [PA], polymerase basic 1 [PB1], and nonstructural [NS] genes) of this natural recombinant virus are attributed to the Eurasian lineage, and phylogenetic analysis showed that those genes are derived from H1N2, H3N1, H3N2, H4N6, H6N2, H10N8, H5N1, and H4N6 avian influenza viruses (AIVs). The amino acid motif of the cleavage site of HA is PSIQSR↓GLF. The sequence analysis will help in understanding the molecular characteristics and epidemiology of the H1N2 influenza virus in sparrows.

## GENOME ANNOUNCEMENT

Avian influenza virus (AIV) is in the influenza A virus family and can cause fatal disease in avian species (1). Hemagglutinin (HA) and neuraminidase (NA) can be used to distinguish different subtypes of AIV. At present, there are at least 17 HA subtypes and 10 NA subtypes of AIV that exist in the world (2–4). It is notable that among the many subtypes of influenza A virus, influenza A H1 and H3 subtypes are currently circulating among humans. The H1 subtype of AIV is transmitted to pigs, and perhaps through pigs as an intermediate host, through gene rearrangement or mutations, influenza virus is in turn transmitted to humans, causing human influenza outbreaks and epidemics (5–7). Therefore, the H1 subtype of AIVs that circulate in avian species should not be ignored.

In this study, a novel strain named A/Sparrow/Guangxi/GXs-1/2012 (H1N2) was first isolated from a domestic sparrow in Guangxi, southern China, in 2012. The nucleotide sequences of this strain were amplified by reverse transcription-PCR (RT-PCR) performed using universal primers (8–10). The amplified products were purified and cloned into the pGEM-T Easy vector (Promega) and were sequenced (TaKaRa, Dalian, China). Sequences were assembled and manually edited to generate the final genome sequence.

The complete genome of A/Sparrow/Guangxi/GXs-1/2012 (H1N2) consists of eight segments, including polymerase (PB2, PB1, PA), HA, nucleoprotein (NP), NA, matrix protein (M), and nonstructural protein (NS) genes. The full lengths of these segments are 2,341, 2,341, 2,233, 1,777, 1,565, 1,466, 1,027, and 890 nucleotides, respectively. The amino acid motif of the cleavage site of HA is PSIQSR↓GLF, which is a typical characteristic of low-pathogenicity AIV. The amino acid residues at the receptor binding site in the HA protein are Q226 and G228, which indicates its AIV-like receptor binding preference. The PB2 protein possesses E627 and D701, which is characteristic of AIV. The PA protein possesses A20, which is antagonistic to antiviral responses. The M2 protein possesses S31, which is not amantadine resistant. E627 and D701 are found in the PB2 sequence, which is characteristic of AIV; A20 in the PA sequence suggests it is antagonistic to antiviral responses; S31 in the M2 protein sequence indicates that the strain has amantadine resistance.

Phylogenetic analysis revealed that the eight genes of A/Sparrow/Guangxi/GXs-1/2012 (H1N2) belong to the Eurasian lineage. The HA gene shows the highest sequence homology (96%) to that from A/ostrich/South Africa/AI2887/2011 (H1N2) (GenBank accession no. JX069105). The NP and MP genes show the highest sequence homologies (97%) to the genes from A/chicken/Pakistan/NARC-16945/2010 (H3N1) (GenBank accession no. HQ165997) and A/duck/Shanghai/C84/2009 (H3N2) (GenBank accession no. JX286592), respectively. The PB2, NA, and PA genes show the highest sequence homologies (98%) to the genes from A/wild duck/Korea/CSM4-28/2010 (H4N6) (GenBank accession no. JX454697), A/duck/Guangxi/GXd-2/2010 (H6N2) (GenBank accession no. JX297589), and A/duck/Guangdong/E1/2012 (H10N8) (GenBank accession no. JQ924792), respectively. In addition, the PB1 and NS genes show the highest sequence homologies (99%) to the genes from A/wild duck/Korea/SNU50-5/2009 (H5N1) (GenBank accession no. JX497766) and A/wild duck/Korea/SH5-26/2008 (H4N6) (GenBank accession no. JX454749). These data will help in understanding the molecular characteristics and epidemiology of the H1 subtype influenza virus in avian species in China.

### Nucleotide sequence accession numbers.

The complete genome sequence of A/Sparrow/Guangxi/GXs-1/2012 (H1N2) was deposited in GenBank under accession no. KF013901 to KF013908.
